# Ambulance referral of more than 2 hours could result in a high prevalence of medical-device-related pressure injuries (MDRPIs) with characteristics different from some inpatient settings: a descriptive observational study

**DOI:** 10.1186/s12873-023-00815-9

**Published:** 2023-04-25

**Authors:** Zhenyu Luo, Sihui Liu, Linhe Yang, Shuyan Zhong, Lihua Bai

**Affiliations:** 1 Guanyuan Central Hospital, Guanyuan, Sichuan China; 2 The First Affiliated Hospital of Soochow University, Suzhou, Jiangsu China

**Keywords:** Medical devices, Pressure injuries, Pressure ulcers, Medical device-related pressure injuries, Ambulance referral

## Abstract

**Background:**

Medical device-related pressure injuries(MDRPI) are prevalent and attracting more attention. During ambulance transfer, the shear force caused by braking and acceleration; extensive medical equipment crowed in a narrow space add external risk factors for MDRPIs. However, there is insufficient research on the relationship between MDRPIs and ambulance transfers. This study aims to clarify the prevalence and characteristics of MDRPI during ambulance transfer.

**Method:**

A descriptive observational study was conducted with convenience sampling. Before starting the study, six PI specialist nurses certified by the Chinese Nursing Association trained emergency department nurses for three MDRPI and Braden Scale sessions, one hour for each session. Data and images of PIs and MDRPIs are uploaded via the OA system by emergency department nurses and reviewed by these six specialist nurses. The information collection begins on 1 July 2022 and ends on 1 August 2022. Demographic and clinical characteristics and a list of medical devices were collected by emergency nurses using a screening form developed by researchers.

**Results:**

One hundred one referrals were eventually included. The mean age of participants was (58.3 ± 11.69) years, predominantly male (67.32%, n = 68), with a mean BMI of 22.48 ± 2.2. The mean referral time among participants was 2.26 ± 0.26 h, the mean BRADEN score was 15.32 ± 2.06, 53.46% (n = 54) of participants were conscious, 73.26% (n = 74) were in the supine position, 23.76% (n = 24) were in the semi-recumbent position, and only 3 (2.9%) were in the lateral position. Eight participants presented with MDRPIs, and all MDRPIs are stage 1. Patients with spinal injuries are most prone to MDRPIs (n = 6). The jaw is the area most prone to MDRPIs, caused by the cervical collar (40%, n = 4), followed by the heel (30%, n = 3) and nose bridge (20%, n = 2) caused by the respiratory devices and spinal board.

**Conclusion:**

MDRPIs are more prevalent during long ambulance referrals than in some inpatient settings. The characteristics and related high-risk devices are also different. The prevention of MDRPIs during ambulance referrals deserves more research.

## Background

As a vital measure of nursing quality and patient safety in healthcare facilities [1], medical device-related pressure injuries(MDRPI) are prevalent and attracting more attention [2–5].

During ambulance transfer, the shear force caused by braking and acceleration [6] and extensive medical equipment crowed in a narrow space add external risk factors for MDRPIs [3, 7]. Some studies explored the relationship between pressure injuries (PI) and ambulance transfers [3, 7]. However, as a specific type of PI identified by the European Pressure Ulcer Advisory Panel (EPUAP) (2019), MDRPI and other PI have different etiologies, its influenced by more medical factors [8]. Research on the prevalence and characteristics of MDRPI in various healthcare settings is necessary [4, 9, 10]. In addition, although the EPUAP stresses the importance of managing MDRPI, no guideline mentions the management of MDRPI during ambulance referrals; the research gap still exists.

China’s emergency medical services system was established in the 1980s [11]. Since then, hospital medical staff have escorted patients by ambulance from primary hospitals to better or unique hospitals for treatment [11, 12]. However, as a vast developing country, emergency services are inequitable, and ambulance transfers can take longer in remote areas [13]. Undoubtedly, with the continuous upgrading of infrastructure in recent years and the establishment of mechanisms such as trauma centers and chest pain centers, there has been a significant reduction in patient transfer times [14, 15]. However, transfers by ambulances from the surrounding counties to the city still take 1½ hours, transfers from more remote villages can take 2–3 h, and to the provincial capital can take even longer, which is long enough for the occurrence of MDRPI [9, 16, 17]. Considering the delays caused by the handover and the uncertainty of traffic conditions, the time patients spend in ambulances could be much longer. However, during the literature search, we found many Chinese studies on pressure injuries in inpatients and emergency department period [10, 18–20], but fail to find out a study on MDRPI in ambulance transfers, and there are differences in the PIs prevalence with some international studies. We hypothesize that several MDRPI occurs during ambulance transfers that are incorrectly identified as occurring in the emergency department period and lead to statistical errors. In addition, even worldwide, studies related to MDRPIs during long-distance referrals in ambulance settings are insufficient and lack detailed evidence (e.g., gender, age, total device days) for guiding clinical MDRPIs prevention [1, 9].

Clarifying the prevalence and characteristics of MDRPI during the ambulance transfer is necessary to improve the quality of care, which is the aim of this study.

## Method

### Study design and setting

The study was approved by the Ethics Committee of Guangyuan Central Hospital and conducted there, the largest referral, medical, and teaching center in the region, which was certificated with the national trauma center, chest pain center, and stroke center in 2016, 2018, and 2019 separately. It has a general hospital and a maternity and pediatric hospital, each with separate emergency departments, with a total of over 2000 referrals per year.

A descriptive observational study was conducted with convenience sampling. All MDRPIs and PIs will be confirmed with the referring medical staff to clarify whether they occurred during or before the referral period. Inclusion criteria were: Admitted to hospital by ambulance; Age > 18 years; Transfer time > 2 h. Exclusion criteria were as follows: MDRPIs or PIs occurring before ambulance referrals; having coagulation disorders (Tends to form petechiae and affect the judgment of MDRPIS); being pregnant; Receiving radiotherapy or chemotherapy within three months; Having dermatitis or burns in MDRPI or PI area; incomplete information. The study was guided by the Sex and Gender Equity in Research (SAGER) guidelines. All patients or legal guardians were informed of the study’s purpose, informed consent was obtained before the start, and all data were de-identified. As no previous studies were found on the prevalence of MDRPI in patients transported by ambulance, the pressure injuries incidence (5.2%) in patients transported by ambulance was selected to measure the sample size [3], a minimum sample size of 76 would be required (z = 1.96, P = 0.052, d = 0.05).

### Data collection

Before the study, six PI specialist nurses certified by the Chinese Nursing Association trained emergency department nurses for three MDRPI and Braden Scale sessions, one hour for each session. The grading of PI and determination of MDRPI is based on [21]; PIs met the criteria “From the use of a device designed and applied for diagnostic or therapeutic purposes. The resulting pressure injury is generally consistent with the pattern or shape of the device” and will be considered MDRPIs; the others will be regarded as other PIs. After training sessions, nurses took an exam with 5 PIs photos and five relevant knowledge questions, correctly answered five questions, and judged all photos with the grade and type of PIs that would be regarded as having adequate knowledge. Data and images of PIs and MDRPIs are uploaded via the OA system (an electronic information system widely used in Chinese hospitals) by emergency department nurses within 1 h of the patient’s arrival and reviewed by these six specialist nurses in 24 h. The information collection begins on 1 July 2022 and ends on 1 August 2022. All PIs and MDRPIs have been treated with body repositioning and decompression dressing timely as they are identified.

Demographic and clinical characteristics and a list of medical devices were collected by emergency nurses using a screening form developed by researchers after consulting the six PI specialist nurses. Gender, age, Body Mass Index (BMI), diagnosis, history of hypertension, and history of diabetes, were collected as demographic information. Clinical characteristics include state of consciousness, Braden scores, transfer time, body position, area of MDRPIs, Medical devices that cause MDRPI, stage of MDRPIs, other PIs, area of other PIs, PI prevention measures, and area of prevention measures.

### Data analysis

Data were entered by SPSS (version 24.0, IBM Corp.). Continuous variables are expressed as mean ± standard deviation (SD); categorical variables are expressed as frequencies and percentages.

## Results

During the data collection, 311 admissions were made by ambulance referral, of which 210 were excluded for not meeting the requirements or not agreeing to participate, and 101 were eventually included (Table 1).

### Demographic characteristics

The mean age of participants was (58.3 ± 11.69) years, predominantly male (67.32%, n = 68), with a mean BMI of 22.48 ± 2.2, 51.48% (n = 52) with a history of hypertension and 53.46% (n = 54) with a history of diabetes mellitus.

### Clinical characteristics

The mean referral time among participants was 2.26 ± 0.26 h, the mean BRADEN score was 15.32 ± 2.06, 53.46% (n = 54) of participants were conscious, 73.26% (n = 74) were in the supine position, 23.76% (n = 24) were in the semi-recumbent position, and only 3 (2.9%) were in the lateral position.

### Prevalence and characteristics of MDRPI

Eight participants presented with MDRPIs (n = 10), of which two participants with spinal injuries presented with two MDRPIs and the rest with one, and all MDRPIs are stage 1. Patients with spinal injuries are most prone to MDRPIs (n = 6). The jaw is the area most prone to MDRPIs, caused by the cervical collar (40%, n = 4), followed by the heel (30%, n = 3) and nose bridge (20%, n = 2), caused by the respiratory devices and spinal board (Table 2). The detailed characteristics of MDRPI cases are shown in Table 3.

**Table 1:** Demographic and clinical characteristics

Characteristics	Frequency (%), Mean ± SD
Age (years)	58.3 ± 11.69
Gender	
Male	68 (67.32%)
Female	33 (32.67%)
BMI	22.48 ± 2.2
History of hypertension	52 (51.48%)
History of diabetes	54 (53.46%)
State of consciousness	
Coma	17 (16.83%)
Drowsiness	16 (15.84%)
Stupor	2 (1.98%)
Conscious	54 (53.46%)
Delirium	6 (5.94%)
Sedation	6 (5.94%)
Transfer time (hours)	2.26 ± 0.26
BRADEN score	15.32 ± 2.06
Body position	
Supine position	74 (73.26%)
Lateral position	24 (23.76%)
Semi-recumbent position	3 (2.9%)

**Table 2:** Prevalence and characteristics of MDRPI

Devices	Frequency (%)	MDRPIs caused (frequency, %).	Area of MDRPIs
Monitoring		0	
SpO2 probe	101 (100%)	0	
ECG	101 (100%)	0	
Blood pressure cuff	101 (100%)	0	
Catheters		1	
Intravenous lines Central venous catheter	97 (96.03%) 1 (0.99%)	0 0	
Urinary catheter	23 (22.77%)	1 (10%)	Left-thigh
Respiratory		2	
Nasal Oxygen	58 (57.42%)	0	
Non-rebreathing mask	14 (13.86%)	0	
Artificial ventilation mask	5 (4.95%)	1 (10%)	Nose bridge
Endotracheal tube	13 (12.87%)	0	
Simple respirator	3 (2.97%)	1 (10%)	Nose bridge
Protective		7	
Spinal board Cervical collar Splints Scoop Stretcher	9 (8.91%) 9 (8.91%) 1 (0.99%) 1 (0.99%)	3 (30%) 4 (40%) 0 0	Heel Jaw

**Table 3:** Detailed characteristics of MDRPI cases

Cases	Gender	Age	Diagnosis	BMI	HTN history (Y/N)	DM history (Y/N)	State of conscious	BRADEN score	Transfer time (hours)		Body position	Area of MDRPIs	Devices related to MDRPIs	Stage of MDRPIs	Other PIs/Stage	Area of PIs	Presence of PI prevention measures/ area
1	Male	74	Spinal injury	22	Y	Y	Drowsiness	12		2.3	Supine	Heel	Spinal board	I	/	/	/
2	Male	56	Spinal injury	23	Y	N	Conscious	15		2.1	Supine	Heel	Spinal board	I	Sacrum/I	/	/
3	Female	27	TBI/Spinal injury	22	N	N	Coma	12		2	Supine	Heel/Jaw	Spinal board/ Rigid cervical collar	I	/	/	/
4	Male	64	TBI	23	Y	N	Coma	14		2.5	Supine	Jaw	Rigid cervical collar	I	/	/	/
5	Female	56	Spinal injury	20	N	N	Conscious	12		2.2	Supine	Jaw	Rigid cervical collar	I	/	/	/
6	Male	33	Spinal Injury	28	N	N	Conscious	15		3	Supine	Jaw/ L-thigh	Adjustable cervical collar/ Urinary catheter	I	/	/	/
7	Male	54	Cerebral hemorrhage	28	Y	Y	Coma	12		2	Supine	Nose bridge	Simple respirator	I	/	/	/
8	Female	43	AECOPD	20	N	N	Conscious	10		2.5	Semi-recumbent	Nose bridge	Ventilation mask	I	/	/	Positive/Sacrum

HTN: hypertension DM: Diabetes mellitus TBI: Traumatic brain injury AECOPD: acute exacerbation of chronic obstructive pulmonary disease.

L-thigh:left-thigh /: not involved.

## Discussion

The results showed that the prevalence and characteristics of MDRPIs during long-term transfers differed from the in-hospital setting. The prevalence of MDRPI in the sample was 7.92%, which is even higher than that in some ICUs, which have been considered the most affected area for MDRPI [1, 4, 9, 22].

For prevalence, eight participants were identified with MDRPIs (n = 10), of which two participants with spinal injuries presented with two MDRPIs and the rest all with one. Patients with spinal injuries are most prone to MDRPIs [23, 24]. The prevalence was 57.14% (4/7), higher than the 20.1% prevalence during hospitalization reported by Ham et al. For those patients, cervical collars are most likely to cause MDRPIs, consistent with Ham et al. However, we also identified spinal boards as a kind of device closely related to MDRPIs (n = 3). In addition, patients with spinal injuries were most likely to have MDRPIs on the jaw and heel in this study, while Ham et al. reported on the back and elbow.

As to devices, ECG and blood pressure cuffs were used at 100%, and IV tubes were used at 96.03%, yet these devices did not cause MDRPIs in long-distance referrals as in ICUs [4, 22]. Similarly, nasal oxygen (57.42% utilization), no re-breathing mask (13.86% utilization), and endotracheal intubation (12.87% utilization) were found to be prone to cause MDRPI in the hospital setting, but not in this study [8, 9, 16]. However, cervical collars and spinal boards were used at 8.91%, leading to four MDRPIs and three MDRPIs, respectively. In addition, this study identified several devices whose association with MDRPIs needed to be adequately studied, e.g., the simple respirator, with a 2.97% utilization rate, caused one case of MDRPIs, which has never been identified before [1, 9]. This device is only an adjunct to the resuscitation process in hospitalized patients and lasts for a short period. However, due to the unevenness of emergency medical resources, some ambulances are not equipped with ventilators, and medical staff can only use simple respirators for extended periods [13]. To prevent MDRPI, it is vital to choose the right size of equipment [8, 25], but for ambulances, narrow spaces make this more challenging to achieve. To cope with unexpected situations, ambulances can only be loaded with kinds of equipment rather than multiple sizes of a device. Case 7 with BMI 28 had a stage I pressure injury on the left elbow caused by a slim stretcher’s handrail, such carriers-induced PIs have not been reported before, but they occur in real situations [1, 9], furthermore, due to the lack of definition of this PI in the guidelines it was not included as researchers failed to make a consensus whether this pressure injury was an MDRPI.

Except for MDRPIs, the prevalence of other PIs was 4.95% (5/101). Considering that only PIs occurring during ambulance referrals were included in this study, the incidence of PIs we found was higher than in a similar study in Australia (Fulbrook et al., 2019). In addition to differences in healthcare resources and economic levels, most PIs or MDRPIs that occur during referral are stage I and often miss reported because nurses believe they will recover quickly [26]. In many cases, ambulance staff is only responsible for the patient’s safety during the referral process. This responsibility handover also confused medical staff and created difficulties in preventing and counting MDRPIs and PIs [7]. In addition, some patients with low BRADEN scores were not received PI prevention, and some patients had only their sacrum protected. The causes of this are complex. [5, 27]. The eight cases presenting MDRPIs showed significant differences in BRADEN scores. Even though MDRPI is defined as a type of PI, it has an entirely different etiology and characteristics, and the ability of the BRADEN score to accurately indicate the risk of MDRPI deserves further study [4].

### Limitation

Firstly this study was conducted in two emergency departments in one city, and if it could be undertaken in more cities, it would undoubtedly increase external validity. Secondly, it could be better if the six PI specialist nurses could re-confirm the judgment of MDRPIs by direct skin inspection rather than photo review. Thirdly, China was experiencing a high temperature during the study period, which may have impacted the referral prevalence of different diseases and may cause a sampling bias.

## Conclusion

MDRPIs are more prevalent during long ambulance referrals than in some inpatient settings. The characteristics and related high-risk devices are also different. The prevention of MDRPIs during ambulance referrals deserves more research.

## Data Availability

The datasets generated and analyzed during the current study are not publicly available due to ethical concerns but are available from the corresponding author on reasonable requests.

## Abbreviations

EPUAP: European Pressure Ulcer Advisory Panel
MDRPI: medical-devices-related pressure injuries
PI: pressure injuries
BMI: Body Mass Index
HTN: hypertension
DM: diabetes mellitus
TBI: traumatic brain injury
AECOPD: acute exacerbation of chronic obstructive pulmonary disease
L-thigh: left-thigh
